# Gut vagal sensory signaling regulates hippocampus function through multi-order pathways

**DOI:** 10.1038/s41467-018-04639-1

**Published:** 2018-06-05

**Authors:** Andrea N. Suarez, Ted M. Hsu, Clarissa M. Liu, Emily E. Noble, Alyssa M. Cortella, Emily M. Nakamoto, Joel D. Hahn, Guillaume de Lartigue, Scott E. Kanoski

**Affiliations:** 1 Human and Evolutionary Biology Section, Department of Biological Sciences, University of Southern California, Los Angeles, California USA; 2 Neuroscience Graduate Program, University of Southern California, Los Angeles, California USA; 3 Department of Psychology, University of Illinois at Chicago, Chicago, Illinois USA; 4 Neurobiology Section, Department of Biological Sciences, University of Southern California, Los Angeles, California USA; 5 The John B. Pierce Laboratory, New Haven, Connecticut USA; 6 Department of Cellular and Molecular Physiology, Yale Medical School, New Haven, Connecticut USA

## Abstract

The vagus nerve is the primary means of neural communication between the gastrointestinal (GI) tract and the brain. Vagally mediated GI signals activate the hippocampus (HPC), a brain region classically linked with memory function. However, the endogenous relevance of GI-derived vagal HPC communication is unknown. Here we utilize a saporin (SAP)-based lesioning procedure to reveal that selective GI vagal sensory/afferent ablation in rats impairs HPC-dependent episodic and spatial memory, effects associated with reduced HPC neurotrophic and neurogenesis markers. To determine the neural pathways connecting the gut to the HPC, we utilize monosynaptic and multisynaptic virus-based tracing methods to identify the medial septum as a relay connecting the medial nucleus tractus solitarius (where GI vagal afferents synapse) to dorsal HPC glutamatergic neurons. We conclude that endogenous GI-derived vagal sensory signaling promotes HPC-dependent memory function via a multi-order brainstem–septal pathway, thereby identifying a previously unknown role for the gut–brain axis in memory control.

## Abstract (web summary)

Feeding-relevant vagal signaling occurs between the gastrointestinal tract and the brain, but it is unclear if this pathway influences cognitive processes. This study shows that endogenous gastrointestinal derived vagal sensory signaling promotes hippocampal-dependent memory function via a multi-order brainstem–septal pathway.

## Introduction

Energy balance and metabolic-relevant communication between the gastrointestinal (GI) tract and the brain is mediated largely by the vagus nerve. Vagal afferent/sensory information is received first in the brain within the medial nucleus of the solitary tract (mNTS) in the caudal brainstem and then relayed to various hindbrain and forebrain regions via ascending neural pathways^1^. Neurons in the hippocampus (HPC), a brain region traditionally linked with learning and memory control and more recently with feeding behavior^2^, are activated by direct vagal nerve stimulation and by GI vagally mediated signals such as mechanical distension of the stomach and intestinal nutrient infusion^3–5^. In addition, rats with selective HPC lesions are impaired in utilizing interoceptive hunger and satiety cues to guide learned anticipatory appetitive outcomes^6^, suggesting that the HPC functionally integrates GI energy balance-relevant cues. Unknown is whether feeding-relevant GI vagal afferent signaling endogenously impacts cognitive and mnemonic processes that are regulated by the HPC.

Consistent with a role for vagal signaling in memory function, vagus nerve stimulation enhances memory^7, 8^, facilitates HPC neurogenesis, and increases HPC expression of brain-derived neurotrophic factor (BDNF)^9, 10^, a neurotrophin that promotes neuronal survival and differentiation, as well as synaptic plasticity^11^. These findings suggest that the vagus nerve promotes neurogenic and neurotrophic signaling. However, these findings involve non-physiological electrical stimulation of the cervical vagus nerve. The endogenous relevance of vagal signaling, especially gut-innervating vagal afferent pathways, to mnemonic and cognitive control is poorly understood. Furthermore, the neural pathways through which vagally mediated energy-state signals are transmitted between the GI tract and hippocampal neurons remains to be fully understood. The mNTS, where GI-derived vagal sensory inputs synapse, sends projections to many brainstem and forebrain sites, but none directly to the HPC^12–14^. Thus, the neural communication between the gut and the HPC must involve a yet unidentified multi-order neural pathway.

**Fig. 1:**
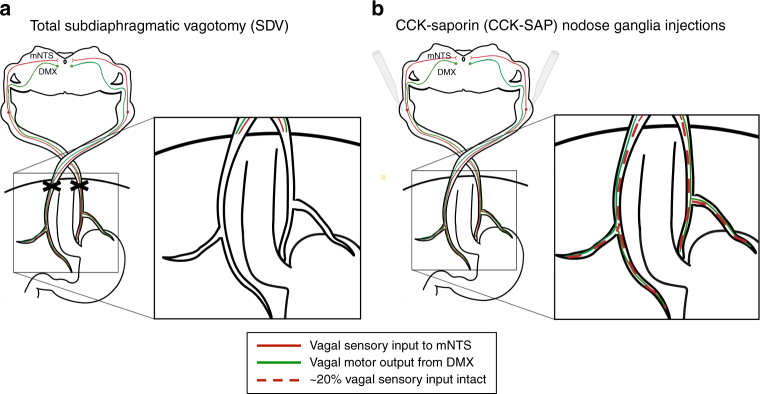
Schematic illustration of subdiaphragmatic vagus nerve ablative disconnection methods. **a** Classic total subdiaphragmatic vagotomy (SDV) surgical method consists of lesioning the dorsal and ventral subdiaphragmatic vagus nerve, eliminating 100% of vagal afferent (sensory) and efferent (motor) signaling below the diaphragm. **b** The novel CCK saporin (CCK-SAP) approach consists of nodose ganglia injections of saporin conjugated to cholecystokinin to specifically ablate ~ 80% of vagal gastrointestinal (GI)-innervating afferent signaling, while leaving 100% of vagal efferent and supradiaphragmatic vagal afferent signaling intact (see ref. ^17^). (DMX dorsal motor nucleus of the vagus nerve, mNTS medial nucleus tractus solitarius). [Cartoon schematic made by authors based on ref. ^42^]

The present study investigated the endogenous role of GI-derived (subdiaphragmatic) vagus nerve signaling on a variety of HPC-dependent memory processes that involve the following: (1) processing of external visuospatial stimuli (i.e., spatial working memory and contextual episodic memory)^15^; (2) discrimination learning based on interoceptive energy status cues (food restriction vs. satiety)^6^; and (3) social transmission of olfactory-related food cues^16^. To dissociate between the role of GI vagal sensory vs. motor signaling on HPC-dependent memory, we utilized total subdiaphragmatic vagotomy (SDV; eliminates all GI vagal afferents and efferents) and a novel rodent surgical approach for selective GI vagal deafferentation in which a SAP conjugated to cholecystokinin (CCK-SAP) is injected into the nodose ganglia (overview of approaches in Fig. 1a, b). This recently established procedure eliminates ~ 80% of GI-derived vagal sensory input to the brain while leaving intact all brain-to-gut vagal motor signaling, and supradiaphragmatic and colonic vagal sensory signaling^17^. Results show that vagal gut–brain sensory signaling is required for hippocampal-dependent learning processes based on external and visuospatial cues, effects accompanied by reduced hippocampal expression of neurotrophic (BDNF) and neurogenic (doublecortin, DCX) markers. Using monosynaptic and multisynaptic virus-based neural pathway tracing methods, we also identified a multi-order pathway connecting the medullary mNTS to the dorsal HPC via medial septum (MS) input to HPC glutamatergic neurons.

## Results

### SDV and CCK-SAP impair contextual episodic memory

**Fig. 2:**
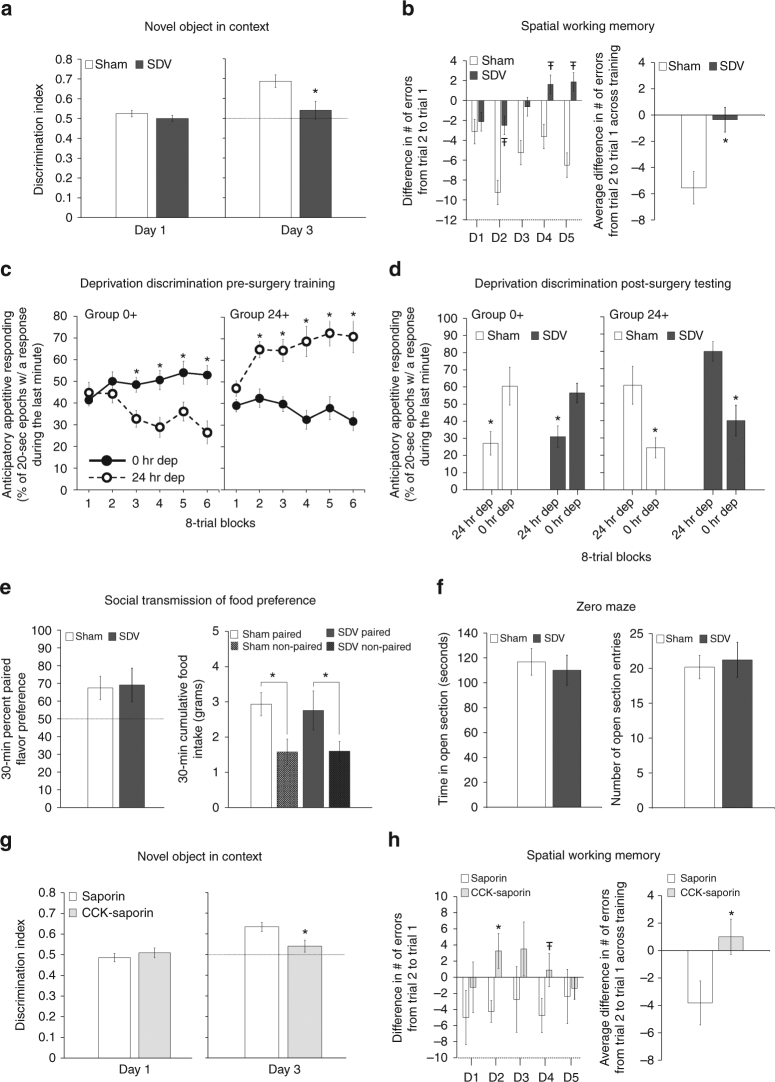
SDV and CCK-Sap impair HPC-dependent contextual episodic and spatial working memory, but not interoceptive, social, or olfactory learning. **a** SDV (*n* = 6) impairs contextual episodic memory relative to controls (*n* = 9); discrimination index on day 1 (habituation) and day 3 (test day) of NOIC testing (repeated-measures ANOVA, F[1,13] = 5.564, *p* = 0.0347; Newman–Keuls’ post hoc, *p* = 0.0047). **b** SDV (*n* = 8) impairs spatial working memory relative to controls (*n* = 8); difference in number of errors from trial 2 (T2) to trial 1 (T1) across individual training days (left) (ANOVA, F[1,14] = 3.626, *p* = 0.0776 (Day 2), F[1,14] = 3.842, *p* = 0.0702 (Day 4), F[1,14] = 3.555, *p* = 0.0803 (Day 5)) and the average T2–T1 errors for each training day in the Barnes maze test (repeated-measures ANOVA, F[1,19] = 6.8565, *p* = 0.0169). **c**, **d** SDV does not impact deprivation intensity discrimination performance; **c** pre-surgery training (Group 0 + , *n* = 16; Group 24 + , *n* = 11; repeated-measures ANOVA, F[1,22] = 135.54, *p* < 0.0001; Newman–Keuls’ post hoc, Group 0 + block 3–6 all *p* < 0.0017, Group 24 + block 2–6 all *p* < 0.000178), **d** and post-surgery testing [mean percent of 20 s epochs of interval magazine entries during the last minute of test session for Group 0 + (sham, *n* = 8; SDV, *n* = 7) and 24 + (sham, *n* = 6; SDV, *n* = 5) under alternating 0 h and 24 h food restriction] (repeated-measures ANOVA, F[1,22] = 80.5115, *p* < 0.00001; Newman–Keuls’ post hoc, all *p* < 0.004). **e** SDV (*n* = 6) does not impact STFP relative to controls (*n* = 9); 30 min percent preference for the socially paired flavored chow and 30 min cumulative food intake (grams) in the STFP test (paired *t*-test, *p* = 0.014 (SDV), *p* = 0.014 (sham)). **f** SDV does not impact anxiety-like behavior; time spent in open arm section (seconds) and number of open section entries during zero maze test for the SDV vs. sham groups. **g** CCK-SAP impairs contextual episodic memory; NOIC discrimination index on days 1 and 3 in CCK-SAP (*n* = 9) and SAP (*n* = 8) control rats (repeated-measures ANOVA, F[1,15] = 6.496, *p* = 0.0223; Newman–Keuls’ post hoc, *p* = 0.0241). **h** CCK-SAP impairs SWM; (T2–T1 error for each individual training day (ANOVA, F[1,13] = 6.824, *p* = 0.0215 (Day 2)) and overall average (repeated-measures ANOVA, F[1,13] = 8.66, *p* = 0.0114) in CCK-SAP (*n* = 8) and SAP (*n* = 7) control rats. (*P < 0.05; ^Ŧ^*P* < 0.08 vs. sham or SAP controls; data are mean ± SEM)

Novel object in context (NOIC) learning is a rodent model of contextual episodic memory. During day 1 of training, SDV and sham groups exhibited similar object discrimination indices (DIs; a measure of exploration time of both objects, Fig. 2a, left), indicating that baseline preference for objects A and B did not differ by group. On test day, SDV animals had impaired contextual episodic memory, demonstrated by a significantly reduced DI relative to sham animals (Fig. 2a, right; a DI above 0.50 means animals spent more time exploring the novel object for the test context). Repeated-measures analysis of variance (ANOVA) analyses across days revealed a significant day × group interaction (F[1,13] = 5.564, *p* = 0.0347), with Newman–Keuls’ post hoc analyses confirming a significant sham vs. SDV group difference on day 3 (*p* = 0.0047) but not on day 1.

Analogous to the SDV and sham group, CCK-SAP and SAP (controls) groups demonstrated similar DIs on day 1 of NOIC training (Fig. 2g, left). On test day, however, the CCK-SAP group had a significantly reduced DI relative to the sham group (Fig. 2g, right). Repeated-measures ANOVA analyses revealed a significant day × group interaction (F [1,15] = 6.496, *p* = 0.0223), with Newman–Keuls’ post hoc analyses confirming a significant SAP vs. CCK-SAP group interaction on day 3 (*p* = 0.0241) but not on day 1. Thus, hippocampal-dependent contextual episodic memory in rats requires intact GI vagal afferent signaling.

### SDV and CCK-SAP impair spatial working memory

We developed a modified Barnes maze procedure to assess hippocampal-dependent spatial working memory in rats in which the spatial location of an escape hole is constant across two consecutive trials per day, but changes each subsequent training day. The index of learning on this task is the difference in the number of errors (exploration of escape holes that do not contain the escape box) from trial 2 to trial 1 for each individual training day. Results show that the SDV group was impaired in spatial working memory performance relative to shams (Fig. 2b, right). Repeated-measures ANOVA analyses of average difference in number of errors from trial 2 to trial 1 revealed significant group main effect across the 5 training days (F[1,19] = 6.8565, *p* = 0.0169). Individual training day analyses showed trends toward a group effect statistical significance on Day 2 (F[1,14] = 3.626, *p* = 0.0776, ANOVA), Day 4 (F[1,14] = 3.842, *p* = 0.0702, ANOVA), and Day 5 (F[1,14] = 3.555, *p* = 0.0803, ANOVA) compared with shams (Fig. 2b, left).

Similar to the SDV and sham groups, the CCK-SAP group was also impaired in this task relative to SAP controls, with repeated-measures ANOVA analyses of average error difference (T2–T1) revealing a significant group main effect across the 5 training days (F[1,13] = 8.66, *p* = 0.0114) (Fig. 2h, left). Individual training day analyses also indicated a significant group effect in error difference (T2–T1) on Day 2 (F[1,13] = 6.824, *p* = 0.0215, ANOVA) relative to sham (Fig. 2h, right). Overall, these findings indicate that spatial working memory in rats is impaired following GI vagal afferent ablation.

### SDV does not affect interoceptive or social learning

Deprivation intensity discrimination learning is a hippocampal-dependent procedure in which rats learned to use interoceptive energy status cues (0 vs. 24 h food restriction) as discriminative stimuli for a forthcoming food reinforcer^6^. Repeated-measures ANOVA over six 8-trial blocks of training (before SDV and sham surgeries) showed that both Group 0 + and Group 24 + learned to respond more (anticipatory food cue entries before food pellet delivery) during the last minute of test sessions under their reinforced compared to non-reinforced food restriction level (Fig. 2c), supported by a significant deprivation state × deprivation group interaction (F[1,22] = 135.54, *p* < 0.0001) and a significant block × deprivation state × deprivation group interaction (F[5,110] = 13.6535, *p* < 0.0001). When analyzing each block individually, Newman–Keuls’ post hoc analyses indicate that Group 0 + responded significantly more under 0 h compared with 24 h food deprivation during blocks 3–6 (all *p*s < 0.0017, Fig. 2c, left), whereas Group 24 + responded significantly more under 24 h compared with 0 h food deprivation during blocks 2–6 (all *p*s < 0.000178, Fig. 2c, right). Testing of deprivation intensity discrimination retention occurred following recovery from SDV and sham surgeries. Results confirmed that SDV lesions had no impact on retention of this type of interoceptive-based discrimination. In Fig. 2d, the effects of total SDV vs. sham surgery on food cup entry during the last minute of each test session performance when both Groups 0 + and 24 + were tested under 0 h and 24 h food deprivation are shown. Repeated-measures ANOVA revealed no significant surgery group effect (F[1,22] = 0.0123, *p* = 0.9126) or deprivation level × surgery group interaction (F[1,22] = 0.1665, *p* = 0.687). Animals in Group 0 + responded significantly more under 0 h than under 24 h food deprivation (Fig. 2d, left) and those in Group 24 + responded significantly more under 24 h than 0 h food deprivation (Fig. 2d, right), regardless of surgical group. These conclusions are supported by Newman–Keuls’ post hoc analyses revealing a significant 0 h vs. 24 h deprivation state interaction for Group 0 + SDV (*p* = 0.0028), Group 0 + sham (*p* = 0.004), Group 24 + SDV (*p* = 0.000589), and Group 24 + sham (*p* = 0.000894). These results suggest that in the absence of GI vagal signaling via SDV, non-vagal cues are sufficient to sustain the learned ability to use interoceptive energy status cues as discriminative stimuli for food reinforcement.

Social transmission of food preference (STFP) is a hippocampal-dependent procedure involving social-based learning using olfactory cues^16^. Percent paired flavor preference at testing was above 50% chance for both sham and SDV groups (Fig. 2e, left). Both sham and SDV group significantly preferred the paired flavored chow to the non-paired flavored chow when tested 24 h after the social interaction (Fig. 2e, right; 30 min cumulative food intake). One-way ANOVA analyses revealed no significant SDV vs. sham group effect for 30 min percent paired flavor preference (F[1,12] = 0.0225, *p* = 0.883). Paired Student’s *t*-test analysis indicated a significant preference for paired vs. non-paired flavored chow for both SDV (*p* = 0.014) and sham (*p* = 0.014) groups. Thus, GI vagal signaling has minimal impact on hippocampal-dependent social olfactory-based learning.

### Neither SDV nor CCK-SAP affect innate anxiety or body weight

The Zero maze procedure is an established rodent model of anxiety-like behavior that is similar to the elevated plus maze procedure. ANOVA revealed no significant surgical group main effect between SDV and shams for time spent in the open section (F[1,19] = 0.0454, *p* = 0.833) (Fig. 2f; left) and number of open section entries (F[1,19] = 4.861, *p* = 0.731) (Fig. 2f; right). Similarly, ANOVA revealed no significant surgical group main effect between CCK-SAP vs. SAP for: time in open section (F[1, 15] = 0.0103, *p* = 0.92) and number of open section entries (F[1,15] = 0, *p* = 1.0; groups had equal means) in the zero maze test of innate anxiety (Supplementary Fig. 1a), as well as center zone distance (F[1,15] = 0.198, *p* = 0.663), number of center zone entries (F[1,15] = 0.6269, *p* = 0.441), and total distance (F[1,15] = 0.1784, *p* = 0.679) in the open field test (Supplementary Fig. 1b). Thus, observed contextual episodic and spatial working memory impairments observed in SDV and CCK-SAP rats are unlikely to be secondary to effects on anxiety-like behavior. Overall, there were three cohorts of SDV and sham animals, and terminal body weights did not differ between surgical groups in any cohort: cohort 1 underwent deprivation intensity discrimination task (sham: 413.54 ± 10.74, SDV: 396.78 ± 11.62; *p* = 0.329, ANOVA), cohort 2 tested in the Barnes task (sham: 391.19 ± 8.33, SDV: 365.09 ± 11.95; *p* = 0.095, ANOVA), and cohort 3 tested in NOIC and STFP tasks (sham: 371.69 ± 7.16, SDV: 354.47 ± 11.42; *p* = 0.199, ANOVA). Similarly, the cohort of SAP and CCK-SAP animals used in this study showed no significant group differences in terminal body weight (SAP: 394.16 ± 11.04, CCK-SAP: 383.28 ± 11.53; *p* = 0.508, ANOVA). Therefore, we conclude that the observed contextual episodic and spatial working memory impairments observed in SDV and CCK-SAP rats are unlikely to be secondary to surgical effects on body weight regulation.

### SDV and CCK-SAP reduce BDNF and DCX in the dHP

**Fig. 3:**
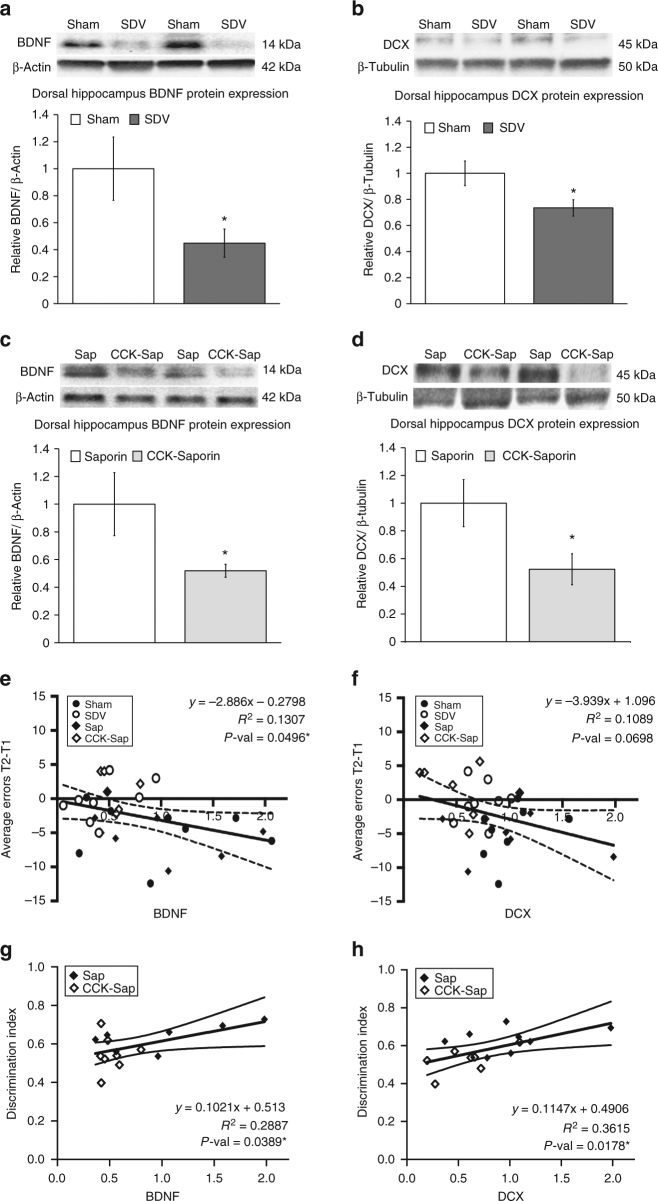
SDV and CCK-SAP reduce BDNF and DCX protein expression in the dorsal HPC and are functionally related to HPC-dependent memory performance. **a**, **b** SDV reduces protein expression of BDNF and DCX (expressed relative to loading control proteins) in dorsal HPC tissue in SDV (*n* = 8) vs. sham-operated control rats (*n* = 8) (ANOVA, F[1,14] = 4.609, *p* = 0.049 (BDNF), F[1,14] = 5.5133, *p* = 0.034 (DCX)). **c**, **d** CCK-SAP-mediated GI vagal afferent ablation (*n* = 8) reduces dorsal HPC BDNF and DCX expression relative to SAP (SAP BDNF, *n* = 7; SAP DCX, *n* = 8) controls (ANOVA, F[1,13] = 4.881, *p* = 0.0457 (BDNF), F[1,14] = 5.494, *p* = 0.034 (DCX). **e**–**h** Linear regression of average number of errors from trial 2 to trial 1 (spatial working memory) and NOIC discrimination index (contextual episodic memory) against relative BDNF and DCX expression reveals a significant negative correlation for BDNF (F[1,28] = 4.211, R2 = 0.1307, *p* = 0.0496) (**e**) with a trend for DCX (F[1,29] = 3.546, R2 = 0.1089, *p* = 0.0698) (**f**). For the novel object in context (NOIC) task of contextual episodic memory, there was a positive correlation between discrimination index and protein expression of BDNF (F[1,13] = 5.277, R2 = 0.2887, *p* = 0.0389) (**g**) and DCX (F[1,13] = 7.36, R2 = 0.3615, *p* = 0.0178) (**h**). (*P < 0.05 vs. controls [sham and/or SAP]; data are mean ± SEM. BDNF brain-derived neurotrophic factor, CCK-SAP cholecystokinin–saporin, DCX doublecortin, SDV subdiaphragmatic vagotomy

Immunoblot analyses from dorsal HPC lysates revealed that total SDV reduced BDNF and DCX protein expression in the dorsal HPC relative to sham controls (Fig. 3a, b), with a significant main effect of surgical group observed for BDNF (F[1,14] = 4.609, *p* = 0.049, ANOVA) and DCX (F[1,14] = 5.5133, *p* = 0.034, ANOVA). Similar to SDV, the CCK-SAP significantly reduced levels of both proteins in the dorsal HPC relative to SAP controls (Fig. 3c, d). This conclusion is supported by one-way ANOVA analyses indicating a significant main effect of surgical group for both dorsal hippocampal BDNF (F[1,13] = 4.881, *p* = 0.0457) and DCX (F [1,14] = 5.494, *p* = 0.034) levels. On the other hand, immunoblot analyses from whole hypothalamic lysates revealed no significant group differences for both BDNF (F[1,13] = 0.26, *p* = 0.619, ANOVA) and DCX (F[1,14] = 0.4955, *p* = 0.493, ANOVA) levels in CCK-SAP vs. control SAP animals (Supplementary Fig. 2 a, b), indicating that GI vagal afferent ablation is unlikely to have systemic brain-wide impact of these neurotrophic (BDNF) and neurogenic (DCX) markers. Hypothalamic tissue was not collected in SDV and sham groups, and therefore analyses for these groups could not be included.

Following Shapiro–Wilk test of normality to confirm that the data were normally distributed [CCK-SAP vs. SAP controls: BDNF, *W* = 0.75377; DCX, *W* = 0.90026; DI, *W* = 0.96494; spatial working memory, *W* = 0.94624; for SDV vs. sham controls: BDNF, *W* = 0.88919; DCX, *W* = 0.92599; DI, *W* = 0.93305; spatial working memory, *W* = 0.91989], linear regression analyses were conducted to examine whether dorsal hippocampal BDNF and DCX protein expression were functionally correlated with HPC-dependent spatial working memory (Fig. 3e, f) and NOIC (Fig. 3g, h) task performance. For Barnes maze, analyses included all groups (sham, SDV, SAP, and CCK-SAP) and revealed that (1) dorsal HPC BDNF levels are negatively correlated with average error difference (T2–T1) (F[1,28] = 4.211, R2 = 0.1307, *p* = 0.0496) and (2) dorsal HPC DCX levels showed a trend toward significant correlation with Barnes performance (F[1,29] = 3.546, R2 = 0.1089, *p* = 0.0698). These results indicate that lower levels of BDNF and DCX are associated with poorer performance in the Barnes task. For NOIC, analyses of SAP and CCK-SAP groups only (as HPC lysates were not collected from SDV and sham rats that performed NOIC) reveal a significant positive correlation for both BDNF (F[1,13] = 5.277, R2 = 0.2887, *p* = 0.0389) and DCX (F[1,13] = 7.36, R2 = 0.3615, *p* = 0.0178) levels with DI, indicating that lower levels of BDNF and DCX are associated with poorer performance in contextual episodic memory.

### CCK activates c-Fos in dCA3 and DG glutamatergic neurons

**Fig. 4:**
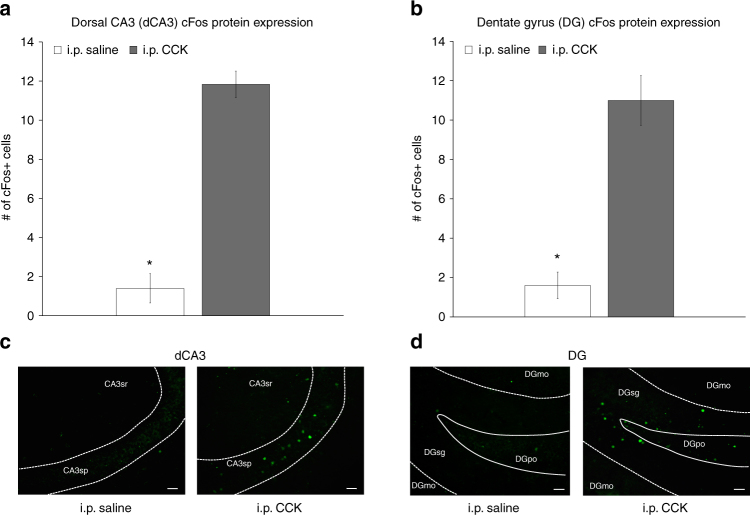
Peripheral administration of CCK activates c-Fos protein expression in the dorsal CA3 (dCA3) and dentate gyrus (DG). Intraperitoneal injections of CCK (*n* = 6) (a vagally mediated gastrointestinal-derived satiation signal) increases the number of c-Fos-immunoreactive (-ir) cells (a marker for neural activation) expressed in the **a** dCA3 and **b** DG vs. saline (*n* = 5) treatment (ANOVA, F[1,9] = 20.236, *p* = 0.001492 (dCA3), F[1,9] = 37.917, *p* = 0.000167 (DG)). Representative images of immunohistochemical staining of c-Fos-ir protein (green) in the **c** dCA3 and **d** DG. Scale bar: 25 μm. (*P < 0.05 vs. i.p. saline controls; data are mean ± SEM.; CCK cholesystokinin, i.p. intraperional, DG dentate gyrus, dCA3 dorsal CA3, CA3sr CA3 stratum radiatum, CA3sp CA3 pyramidal layer, DGmo dentate gyrus molecular layer, DGpo DG polymorph layer, DGsg DG granule cell layer)

**Fig. 5:**
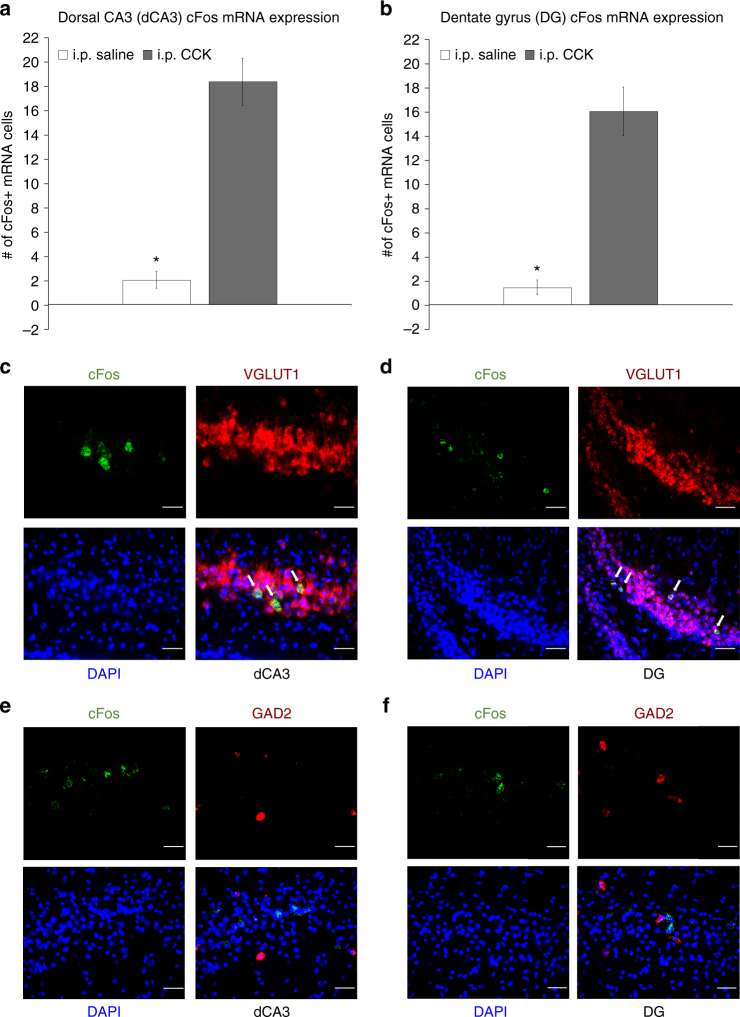
Peripheral administration of CCK activates c-Fos mRNA expression in dCA3 and DG hippocampal glutamatergic neurons. Number of c-Fos-labeled (c-Fos + ) cells for mRNA (fluorescent in situ hybridization) expressed in the **a** dCA3 and **b** DG following i.p. administration of CCK (*n* = 6) or saline (*n* = 5) (ANOVA, F[1,9] = 53.093, *p* = 0.000046 (dCA3), F[1,9] = 40.496, *p* = 0.000131 (DG)). Approximately 93% and 94% of c-Fos + cells in the dCA3 and DG were VGLUT1 + (respectively) following i.p. CCK treatment, whereas only 4% and 8% of c-Fos + cells in the dCA3 and DG were GAD2 + (respectively) following i.p. CCK. **c**, **d** Representative images show c-Fos mRNA (green) and VGLUT1 (red) or GAD2 (**e**, **f**) (red) mRNA expression in dCA3 (**c**, **e**) and DG (**d**, **f**) cell bodies following i.p. CCK (DAPI nuclear stain; blue). Scale bar: 25 μm. (Arrows, co-expression of c-Fos/VGLUT1 mRNA cells; *P < 0.05 vs i.p. saline controls; data are mean ± SEM. CCK cholesystokinin, dCA3 dorsal CA3, DG dentate gyrus, i.p. intraperional)

Intraperitoneal (i.p.) injections of CCK-8 (8 μg/kg) increased the number of c-Fos protein immunoreactive cells expressed in the dorsal CA3 (dCA3; F[1,9] = 20.236, *p* = 0.001492, ANOVA) (Fig. 4a, c) and dentate gyrus (DG; F[1,9] = 37.917, *p* = 0.000167, ANOVA) (Fig. 4b, d) relative to i.p. saline treatment. In addition, i.p. CCK injections increased the number of labeled cells for c-Fos messenger RNA (fluorescent in situ hybridization, FISH) expressed in the dCA3 (F[1,9] = 53.093, *p* = 0.000046, ANOVA) (Fig. 5a) and DG (F[1,9] = 40.496, *p* = 0.000131, ANOVA) (Fig. 5b) relative to saline treatment, with 93.11% and 94.35% of c-Fos mRNA-positive cells in dCA3 (Fig. 5c) and DG (Fig. 5d) being VGLUT1 positive, respectively, and only 4.29% (Figs. 5e) and 8.44% (Fig. 5f) of c-Fos mRNA-positive cells being GAD2 mRNA positive, respectively.

### Medial septum connects mNTS neurons to the dorsal HPC

**Fig. 6:**
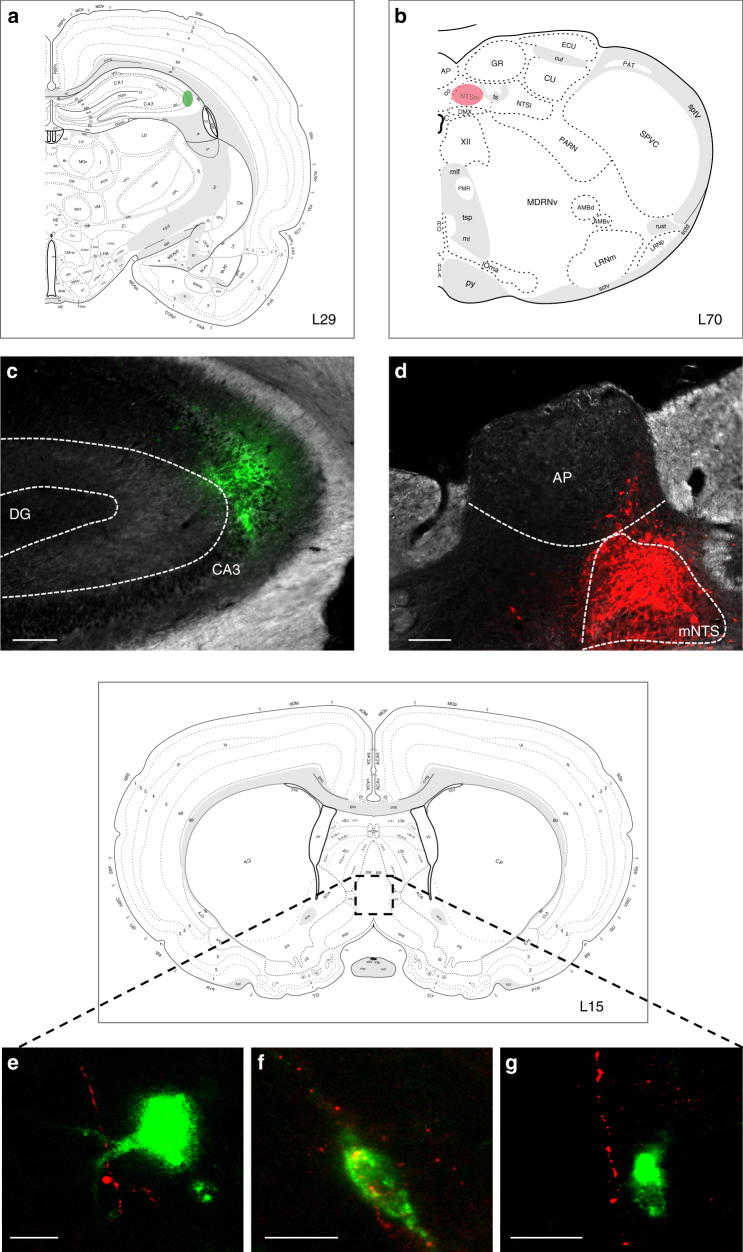
Co-injection monosynaptic neural pathway tracing strategy identifies the medial septum (MS) as a relay region connecting the mNTS to the dHPC (CA3). Schematic representative injection sites in **a** dCA3 and **b** mNTS in Swanson Atlas level 29 and 69, respectively. **c** Unilateral iontophoretic dCA3 injection site of the retrograde tracer, CTB-488 (green). Scale bar: 100 μm. **d** Ipsilateral and unilateral iontophoretic mNTS delivery of the anterograde viral tracer, AAV1-TurboRFP (red) (*n* = 3 double hits, *n* = 11 controls). Scale bar: 500 μm. **e**, **f**, **g** RFP-ir axons from the mNTS in apposition to CTB-488-ir cell bodies from dCA3 in the MS (images made by authors and adapted from Swanson Atlas level 15^68^). Scale bar: 10 μm. (AP area postrema, DG dentate gyrus, mNTS medial nucleus tractus solitarius)

The medial nucleus tractus solitarius (mNTS) is the first central nervous system (CNS) site to receive GI-derived vagal sensory input; however, the mNTS does not communicate monosynaptically with the HPC^13^. To identify regions of possible relay between the mNTS and HPC, a combination of retrograde and anterograde pathway tracing was used: unilateral iontophoretic injections of a retrograde pathway tracer targeted to the dCA3 (cholera toxin subunit B (CTB) AlexaFluor 488 (AF488) conjugated) (Fig. 6a, c) were combined with ipsilateral iontophoretic injections of an anterograde pathway tracer targeted to the mNTS (AAV1-hSyn-TurboRFP-WPRE-rBG) (Fig. 6b, d). Red fluorophore (red fluorescent protein (RFP) and Cy3 following immunohistochemistry (IHC)) anterogradely labeled axons originating from the mNTS were found in apposition to green fluorophore (AF488 following IHC) labeled cell bodies in the MS that were retrogradely labeled from dCA3 in each of the three animals that were confirmed as double hits in both injection sites (representative appositions in Fig. 6e–g), whereas such appositions were not observed in various control animals (*n* = 11) in which either (or both) injection site(s) were either undeterminable or adjacent to the intended target.

**Fig. 7:**
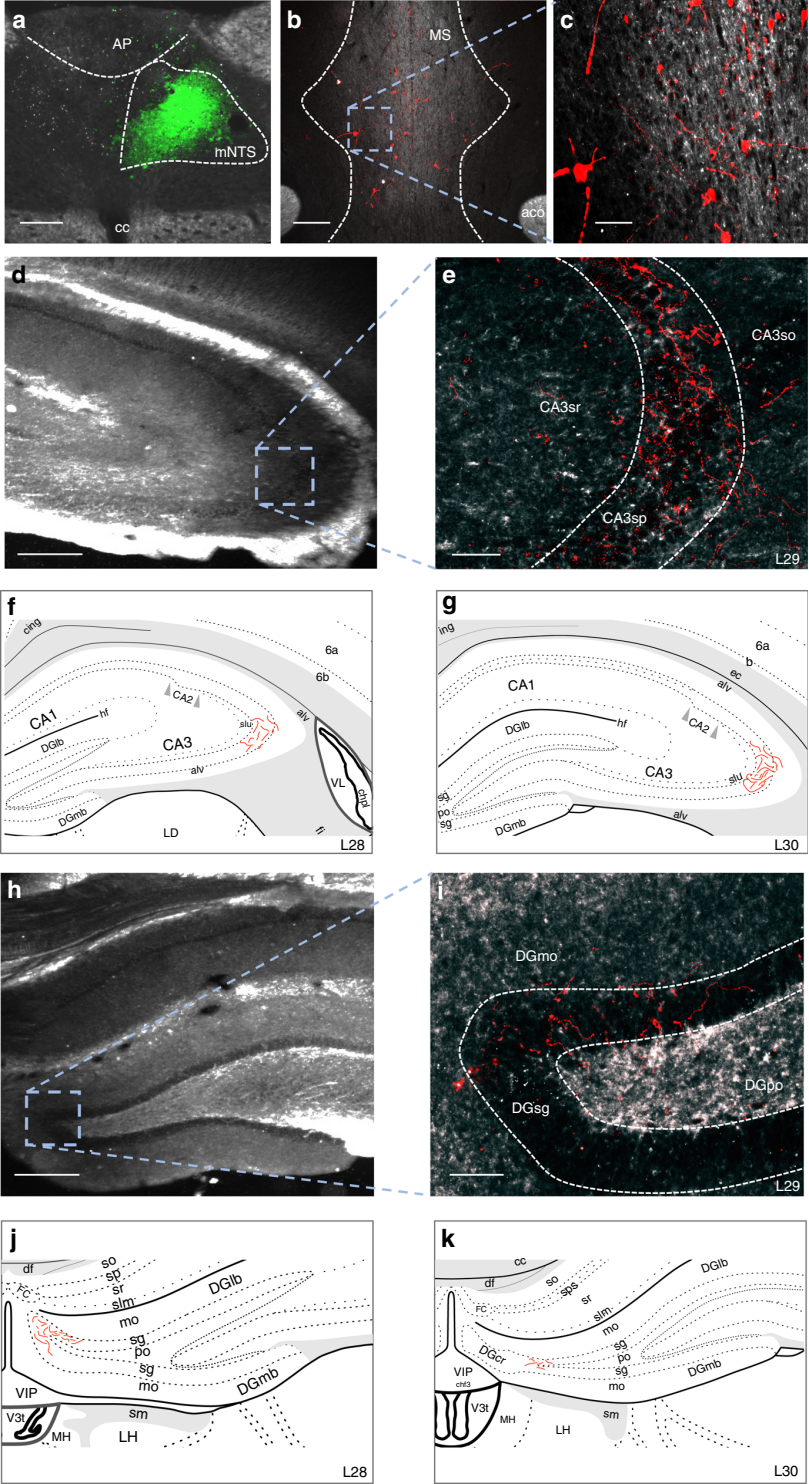
Multisynaptic viral tracing approach reveals MS neurons that receive monosynaptic input from the mNTS directly project to the dHPC (dCA3 and DG). **a** Unilateral iontophoretic co-injection of AAV2/1-hSyn-Cre and CTB (CTB-ir in green; to confirm injection site placement) in the mNTS (*n* = 3 double hits, *n* = 13 controls), which drives Cre expression in second-order (but not third-order) neurons based on synaptic virion release from first-order axon terminals^18^. Scale bar: 100 μm. **b**, **c** A 200 nl pressure injection site of a Cre-dependent anterograde tracer (AAV1-CAG-FLEX-TdTomato) in the MS Scale bar: **b** 200 μm, **c** 50 μm. Axon terminal fields in the **d**, **e** dCA3 and **h**, **i** DG of MS neurons that receive direct input from mNTS. Scale bar: **d**, **h** 250 μm, **e**, **i** 50 μm. A schematic representation of dCA3 (**f**, **g**) and DG (**j**, **k**) axon terminal field distribution (Made by co-author and adapted from Swanson atlas level 28–30^68^). (aco anterior commissure, AP area postrema, CA3sr CA3 stratum radiatum, CA3sp CA3 pyramidal layer, DGmo dentate gyrus molecular layer, DGpo DG polymorph layer, DGsg DG granule cell layer, mNTS medial nucleus tractus solitarius, MS, medial septum)

To further support this multi-order pathway, we utilized a novel dual-synaptic virus-based pathway tracing approach to examine whether mNTS neurons synpatically communicate to the HPC via a MS relay pathway^18^. The AAV2/1-hSyn-Cre drives Cre expression in first-order neurons infected at the injection site, as well as in second-order (but not third-order) neurons based on virion release from first-order axon terminals^18^. A unilateral iontophoretic co-injection of AAV2/1-hSyn-Cre (and CTB-488 to confirm injection site) targeted to the mNTS (at the level of the area postrema; Fig. 7a) was followed by a pressure injection of AAV1-CAG-FLEX-TdTomato (a Cre-dependent anterograde tracer) targeted to the MS (Fig. 7b, c). Results revealed robust axon terminal fields in the dCA3 (Fig. 7d, e) and DG (Fig. 7h, i) for each of the three animals that were confirmed as double hits in both injection sites. A schematic of the rostral-caudal distribution of axon terminal fields in the dCA3 (Fig. 7f, g) and DG (Fig. 7j, k) from a representative double hit animal are displayed. Axonal labeling in the dHPC was not observed for rats (*n* = 13) in which either the mNTS or MS injection was absent or adjacent to the targeted region. Overall, these results indicate the presence of synaptic connections to the dHPC from MS neurons that receive direct input from the mNTS.

## Discussion

Our results reveal that GI-derived vagal sensory signaling endogenously promotes hippocampal-dependent learning and memory function in rats. Both classic nonselective (SDV; eliminates all GI vagal afferents and efferents) and novel sensory-selective (CCK-SAP approach; eliminates ~ 80% of upper GI vagal afferents) ablative methods of GI vagal disconnection impaired hippocampal (HPC)-dependent memory processes, including spatial working memory and contextual episodic memory. Moreover, both ablative methods reduced expression of neurogenic (DCX) and neurotrophic (BDNF) markers in the dorsal HPC that promote neurogenesis and plasticity, and expression of these markers in the HPC were correlated to both spatial working memory and contextual episodic memory. We further investigated HPC involvement in GI vagal sensory signaling by analyzing neuronal activation in the HPC in response to the GI-derived vagally mediated satiation signal, CCK. Expression of c-Fos in response to peripheral CCK was robust in the HPC, predominantly present in glutamatergic neurons in the dorsal HPC CA3 and DG. To identify multi-order neuronal pathways by which GI vagal sensory signaling communicates to the HPC, we employed multiple innovative multi-order neural pathway tracing strategies. These approaches identified the MS as a relay region between the mNTS (first site of vagal sensory input to the CNS) and the dorsal HPC (CA3 and DG). Overall, these results reveal a novel role for gut-to-brain communication in the control of learning and memory function and identify a putative neuronal pathway through which this communication may occur.

Previous work using electrical vagus nerve stimulation approaches involving non-physiological stimulation of the cervical vagus nerve (and is therefore not selective to GI vagal signaling) revealed improved word-recognition memory in humans following vagus nerve stimulation^8^. Similarly in rodents, vagus nerve stimulation improved retention of inhibitory-avoidance memory^7^ and facilitated extinction of conditioned fear^19^. Our data expand these findings by establishing a physiological role for vagal sensory signaling, specifically that originating in the GI tract, in HPC-dependent memory function. These findings may have clinical relevance in relation to current treatments for obesity that involve disruptive manipulation of the vagus nerve, such as bariatric surgeries (e.g., RYGB, vertical sleeve gastrectomy)^20^ and chronic electrical disruption of vagal nerve signaling (e.g., VBLOC^21^).

Our results support the notion that gut to brain vagally-mediated communication has an important role in protecting against neurodegenerative disorders (e.g., Alzheimer’s disease). For example, human patients with Alzheimer’s disease showed improved cognition following 6 months of chronic cervical vagus nerve stimulation treatment^22^. At a mechanistic level, recent studies have indicated a functional role for the gut microbiome in regulating the interaction between GI signaling and cognition^23^. For example, in germ-free mice that lack a microbiome, hippocampal BDNF levels are reduced^24^, and these effects are associated with cognitive dysfunction^25^. In addition, total SDV impairs the ability of butyrate, a short-chain fatty acid synthesized by colonic microbiota, to reduce appetite and prevent diet-induced obesity^26^, suggesting a critical need for preservation of vagal nerve signaling in the microbiome–gut–brain axis. Given that butyrate promotes hippocampal neurogenesis and memory function^27^, one possible mechanism linking GI vagal afferent signaling to HPC function is via gut microbial interactions with short-chain fatty acid production.

We examined the effects of SDV-mediated GI vagal ablative disconnection on a variety of HPC-dependent mnemonic and cognitive processes that rely on the utilization of different cues (external, interoceptive, social, olfactory), and that differ in their reliance on discrete HPC subregions. Of the various memory procedures assessed, SDV significantly impaired spatial working memory (Barnes maze procedure) and contextual episodic memory (NOIC), whereas performance in appetitive learning based on internal energy-state cues (deprivation intensity discrimination) and social transmission of food-related (olfactory) cues (STFP) was preserved. The lack of group differences between SDV and control animals for both deprivation intensity discrimination and STFP learning could be due to differential effects of vagal nerve signaling on two functionally and anatomically-independent subregions of the HPC, as studies have shown the dorsal (septal in rodents, posterior in primates) HPC is associated predominantly with visuospatial-based exteroceptive memory, whereas the ventral (temporal in rodents, anterior in primates) HPC is associated with conditioned appetitive and anxiety-like behaviors^2, 28, 29^. The contextual episodic (NOIC) and spatial working memory (Barnes maze) tasks used in the present study rely on the integration of visuospatial external environmental cues. Conversely, deprivation intensity discrimination and STFP rely on conditioned appetitive cues (internal energy-state and social cues, respectively). Deprivation intensity discrimination learning is impaired by lesions to complete, dorsal, or ventral HPC^6, 29^, whereas STFP is primarily linked with ventral hippocampal substrates^16^. Thus, overall our data suggest gut-derived vagal signaling promotes memory involving external environmental cues (that may rely predominantly on the dorsal HPC), while having less impact on conditioned appetitive and anxiety-like behaviors (that may rely predominantly on the ventral HPC). Considering these findings, we hypothesize that interoceptive processing for deprivation intensity discrimination learning is sustained in the absence of GI vagal input, and may therefore primarily involve circulating endocrine and other metabolic signals that communicate to the HPC. Consistent with this framework, injections of the gut-derived hunger hormone, ghrelin, into the ventral but not dorsal HPC increase food intake^30^, and intracerebroventricular ghrelin injections generalize to 24 h food restriction in non-restricted rats trained in the deprivation discrimination task^31^. However, one limitation of the design with regards to comparing across these different tasks is that the learning of interoceptive and social cue-based tasks occurred at different times post surgery. Further, although the experimental design was consistent across surgical groups with regards to time between surgery and behavioral testing, complete counterbalancing of behavioral experiments that involved multiple comparisons was not employed and is therefore a limitation of the study.

Episodic memory, or memory of a specific event, is HPC-dependent and was impaired by SDV (NOIC procedure). Episodic memories are important for the control of feeding behavior and energy balance, as they are critical for animals to remember aspects about where (food location), what (nutritive vs. adverse postingestive consequences), and when eating occurs. Consistent with this notion, experimental manipulations in human subjects designed to disrupt episodic memory during feeding increase hunger ratings and food intake at a subsequent eating episode^32, 33^. Similarly in rats, disruption of episodic meal-related memory via postprandial dorsal hippocampal infusion of muscimol decreases the latency to start the post-infusion meal and increases the size of the post-infusion meal^34^. From an evolutionary perspective, the physiological role of GI-derived vagal sensory signaling in HPC-dependent memory may normally function to enhance episodic memory for eating occasions, as GI vagal sensory signaling is most heavily engaged during feeding. Moreover, given that it is advantageous to remember the physical location of the food source to inform future foraging behavior, the visuospatial external environment is likely to be a critical component of episodic meal-related memories. From this perspective, GI vagal sensory signaling during meal taking represents an advantageous biological survival mechanism that promotes meal-related episodic memory to facilitate future feeding.

Based on the impaired spatial working and contextual episodic memory following SDV (that eliminates both GI sensory and motor signaling), we next demonstrated that a novel selective GI vagal sensory ablation surgical method (CCK-SAP) also impaired both of these HPC-dependent memory processes. These findings suggest that GI vagal sensory/afferent (and not motor/efferent) signaling promotes HPC-dependent memory. The CCK-SAP approach selectively eliminates ~ 80% of GI-derived vagal afferent signaling below the diaphragm^17^, which differs from the established surgical subdiaphragmatic deafferentation procedure (SDA), which involves cutting the vagal sensory (afferent) rootlets unilaterally near the brainstem interface and then ablating the contralateral subdiaphragmatic vagal trunk. SDA eliminates all GI vagal to mNTS sensory signaling, while leaving 50% of supradiaphragmatic sensory and 50% of vagal motor (efferent) signaling intact^35^. Previous work has shown that SDA has no effect on novel object recognition memory or working memory in a non-spatial alternation task^36^. In contrast, here we show that both SDV and CCK-SAP impairs a similar NOIC task. Although novel object recognition relies on visual object recognition memory (i.e., the animal must remember, which object it has previously seen), the NOIC test relies on external visuospatial and/or contextual memory (i.e., animal must remember the location in which it previously encountered an object). Based on previous work, it remains controversial whether the HPC is critically involved in novel object recognition learning, but rather mediates recognition memory when it requires remembering that a stimulus occurred in a certain place or time^37^. The perirhinal cortical area, on the other hand, is more strongly linked novel object recognition memory, as lesions to this brain region impair the ability of animals to discriminate between familiar and novel objects^37–39^. Moreover, rats presented with novel versus familiar objects or pictures have increased c-Fos expression in perirhinal cortex but not HPC, whereas the HPC, but not perirhinal cortex shows increased c-Fos expression in response to novel spatial arrangements of familiar objects^40, 41^. Whether or not the SDA approach, like SDV and CCK-SAP approaches, impairs spatial and/or contextual-based HPC-dependent memory requires further study.

STFP and NOIC involve consumption of novel foods and exposure to novel objects, respectively, which are also used to assess anxiety-like behavior in rodents (neophobia). Thus, we tested both SDV and CCK-SAP rats in the zero maze anxiety test. Moreover, we also tested the CCK-SAP animals in an additional anxiety-relevant task, the open field test. Results revealed no significant group differences in either SDV or CCK-SAP groups relative to controls in these anxiety-related tests. These results differ from a previous study that demonstrated reduced innate anxiety-like behavior in SDA rats^42^. These effects could be due to procedural differences, as unlike the individually housed rats in the present study, the rats in the previous study were group-housed, which has been shown to demonstrate a decrease in mean arterial blood pressure and heart rate relative to isolated (single-housed) male and female Sprague–Dawley rats^43, 44^. In addition, we tested the SDV and CCK-SAP groups in an elevated zero maze, whereas the SDA rats in the previous study were tested in an elevated plus maze. Although both the zero maze and the elevated plus maze are well accepted tests for measuring anxiety-like behavior in rodents, untreated/normal rats show increased exploration time of the open areas in the elevated plus vs. zero maze, potentially due to the time spent in the center (neutral) region of the plus maze, to which these is no equivalent in the zero maze^45, 46^. Although a more extensive analysis of anxiety was conducted in this previous study (we did not perform the food neophagia test), it is worth noting that the elimination of 50% of the vagal afferents above the diaphragm via SDA could be a potential reason for different innate anxiety-like effects of SDA relative to SDV and CCK-SAP animals, as the supradiaphramatic vagal afferents are preserved in these two latter approaches. Consistent with this possibility, optogenetic activation of nonselective vagal afferents (including those innervating cardiac systems) robustly reduces heart rate in mice^47^, and transgenic overexpression of angiotensin-(1–7) in mice chronically reduces heart rate and is accompanied by reduced anxiety-like behavior^48^. Future research is needed to directly examine the role of different vagal afferent neuron populations in anxiety-like behavior.

The mNTS, where GI vagal sensory input arrives in the CNS, does not send direct projections to the HPC^13^. Here we show that peripheral injections of CCK, a vagally mediated GI-derived satiation signal, significantly increased c-Fos protein and mRNA expression in the dorsal HPC CA3 and DG, suggesting a multi-order connection between mNTS and the dHPC. We utilized a monosynaptic co-injection neural pathway tracing method to identify the MS as a possible relay region connecting mNTS to the HPC^49^. As this method does not determine synaptic communication between immunoreactive cell bodies (back-labeled from the CA3) in apposition to axon terminals emanating from the mNTS, we employed a dual-synaptic viral-mediated anterograde tracing method and confirmed the MS as a mNTS to HPC relay region^18^. Although the dual-synaptic AAV1-Cre can be transported in both the anterograde and retrograde (from axon terminals) direction^18^, we limited the application to a pathway where there are no reciprocal connections between targeted pre- and postsynaptic regions. While the mNTS projects to the MS, descending MS projections do not project to the caudal brainstem^50^. Previous studies have established a role of septal cholinergic function in HPC-dependent learning and memory processes. Future work could investigate whether cholinergic signaling is critical in regulating GI vagal modulation of HPC-dependent spatial working and episodic memory function.

Collective results from the present study demonstrate that endogenous vagal afferent signaling from the GI tract regulates HPC-dependent contextual episodic and spatial working memory, potentially by driving the expression of memory-related neurotrophic (BDNF) and neurogenic (DCX) signaling pathways. These findings compliment and expand previous work using cervical vagus nerve stimulation, as well as a recent report showing that total SDV in mice reduces HPC DCX^51^. We further identify the MS as a likely relay connecting the gut to glutamatergic dorsal HPC neurons. Our results further expand previous work by revealing that gut-derived signals, either vagal^36, 42^ or endocrine^52^, interact with higher-order brain regions to regulate memory and cognition. These findings have direct clinical relevance, as common bariatric surgeries partially denervate vagal signaling^20, 53, 54^ and chronic vagus nerve blockade (VBLOC) was recently Food and Drug Administration-approved for obesity treatment^21^. Future studies investigating the neuroendocrine and neural pathways conveying energy-relevant signals between the GI tract and the HPC (and other regions of the telencephalon and cortex) will provide additional insight into the complex role of gut-to-brain communication in cognitive control.

## Methods

### Animals

Male Sprague–Dawley rats (Envigo; 320–450 g on arrival) were individually housed with ad libitum access (except where noted) to water and chow (LabDiet 5001, LabDiet, St. Louis, MO) on 12 h:12 h light/dark cycle (lights on at 08:00 h). All procedures involving animals were approved by the University of Southern California Institute of Animal Care and Use Committee.

### Total SDV

Rats were habituated to liquid diet (Research Diets; AIN76A) for five days before surgery. Following a 24 h fast and under ketamine (90 mg/kg), xylazine (2.7 mg/kg), and acepromazine (0.64 mg/kg) anesthesia and analgesia (Metacam 2 mg/kg), the trunks of the subdiaphragmatic vagus nerve were transected as described previously^55^. A midline abdominal incision was made and then the stomach was retracted caudally and the liver was retracted cranially to expose the esophagus. The dorsal and ventral branches of the vagus were then dissected from the esophagus. Each vagal branch was ligated twice with a surgical thread at an interval of 1–2 cm, and then cauterized between the ligatures. In sham surgeries, the trunks were exposed but the vagus nerve was not ligated or cauterized. The incision was then closed with running sutures along the abdominal wall and stop sutures along the skin. Rats were allowed to recover until liquid diet intake stabilized (at least 1 week) and were then returned to a standard chow diet. After behavioral testing, SDV was verified functionally with i.p. CCK-induced food intake reduction as described^56, 57^. Briefly, the functional verification consists of analysis of food intake following i.p. CCK-8 (2 μg/kg; Bachem) or saline injections (treatments given counterbalanced on separate days) after an overnight fast. SDV rats were included in the statistical analysis if CCK treatment resulted in a < 30% reduction of their food intake, as described^57, 58^. Of the three separate cohorts of rats that underwent SDV surgery and subsequent behavioral testing, four animals were removed from deprivation intensity discrimination analyses and one animal was removed from NOIC analyses based on these criteria.

### CCK-SAP nodose ganglia injection

CCK-SAP targets CCK receptor expressing cells, which are localized on vagal afferent neurons that specifically innervate the upper GI tract^59, 60^. As recently confirmed, CCK-SAP injection in the nodose ganglia (vagal afferent cell bodies) selectively eliminates ~ 80% of GI-derived vagal afferent signaling, while preserving colonic and supradiaphragmatic vagal sensory pathways, as well as all vagal efferents^17^. Twenty-four hours before surgery, rats were given 15 ml of condensed milk in addition to their normal ad libitum access to chow and water, and were fasted before lights went off (18:00 h). Twenty minutes before surgery, rats received an i.p. injection of atropine sulfate (0.05 mg/kg) and carprofen (5.0 mg/kg; Henry Schein), and then anesthetized with a ketamine (90 mg/kg), xylazine (2.7 mg/kg), and acepromazine (0.64 mg/kg) cocktail. A midline incision was made along the length of the neck. The vagus nerve was separated from the carotid artery with Graefe forceps until the nodose ganglion was visible and accessible. A glass capillary (20 μm tip, beveled 30° angle) attached to a micromanipulator was used to position and puncture the nodose ganglion and 1 µl volume of CCK-SAP (250 ng/µl) or SAP (250 ng/µl) was injected with a Picospritzer III injector (Parker Hannifin) at two sites: 0.5 µl rostral and 0.5 µl caudal to the laryngeal nerve branch. The same procedure was performed for both nodose ganglia on either side before the skin was closed with sterile and stop sutures along the skin. Rats were returned to their home cage and deprived of water for 6 h and food overnight. The post-op care was optimized to avoid excessive weight loss post surgery and increase survival rate, as previously described^61^. The schedule was as follows: Day 1 post-op rats received carprofen (5.0 mg/kg; SQ) and were given ad libitum access to condensed milk; day 2 post-op rats received mash (10 g powdered chow mixed with 20 ml condensed milk diluted as described above); day 3 post-op rats received mash and solid chow pellets; day 4 and onwards, rats were given ad libitum access to chow. After behavioral testing, CCK-SAP was verified functionally with i.p. CCK-induced food intake reduction. The functional verification modeled that of the SDV approach described above and that published for CCK-SAP in^17^. Based on this verification test, four CCK-SAP rats were removed from all analyses.

### General research design

Three separate cohorts of rats underwent SDV (or sham) surgery and subsequent behavioral testing (described below), beginning 7 days post surgery. Cohort 1 underwent deprivation intensity discrimination training (described below) in which rats were assigned to one of two groups (group assignment matched based on body weight): Group 0 + (*n* = 16) or Group 24 + (*n* = 11). After asymptotic discrimination was reached, animals were then subdivided (matched based on body weight and performance over the last 8-trial block of training) into two additional groups to receive SDV (Group 0 + , *n* = 7; Group 24 + , *n* = 5) or sham (Group 0 + , *n* = 8; Group 24 + , *n* = 6) surgery 4 days after the last training day. After 7 days of post-surgery recovery, the animals were tested on deprivation discrimination performance for one 8-trial block (see Supplementary Table 1). Seven days after deprivation discrimination testing, animals in Cohort 1 were tested in the zero maze task. Cohort 2 (SDV *n* = 8; sham *n* = 8) was tested in the spatial working memory task (5 days, described below) 7 days post surgery. Five to 6 days later, dHPC tissue was harvested from Cohort 2 for immunblot analyses of BDNF and DCX (SDV, *n* = 8; sham, *n* = 8) (see Supplementary Methods). Cohort 3 (SDV *n* = 6, sham *n* = 9) was tested in the NOIC (5 days) task 14 days post surgery, followed by STFP 21 days post surgery (STFP; 3 days). For STFP (described below), the SDV and sham groups were observers, whereas demonstrator rats (non-operated, *n* = 8) were housed in a separate room from the observers. Based on results from our SDV experiments, CCK-SAP (*n* = 9) and SAP (control, *n* = 8)] rats were tested in the Barnes task (beginning 7 days post surgery; matching the timeline of post surgery SDV Barnes testing) followed by the NOIC task (beginning 14 days post surgery; matching the timeline of post surgery SDV NOIC testing). Seven days after NOIC testing, animals were tested in the zero maze task followed by the open field task 3 days later. Five to 6 days later, dHPC tissue was harvested for immunblot analyses of BDNF (CCK-SAP, *n* = 8; SAP, *n* = 7) and DCX (CCK-SAP, *n* = 8; SAP, *n* = 8) (see Supplementary Methods; see Supplementary Fig. 3 for uncropped scan of CCK-SAP vs. SAP BDNF blot). Groups were assigned matched according to body weight at the beginning of each experiment. For all video analyses for behavioral variables, experimenters were blinded to group assignments of the animals.

### Novel object in context

NOIC incorporates object recognition within a specific context, a test of contextual episodic memory. Procedures followed those previously described^15, 62^. Rats undergo 2 days of habituation: half of the rats (counterbalanced within surgical groups) are able to freely explore Context 1, a semi-transparent box (15 in W × 24 in L × 12 in H) with orange stripes, for 5 min, whereas the other half are habituated to Context 2, a gray opaque box (17 in W × 17 in L × 16 in H). The following day, groups are switched and habituated to the other context under the same conditions. Twenty-four hours later, on day 1 of NOIC, each animal is exposed to two distinct objects: a 500 ml jar filled with blue water (object A) and a square glass container (object B) in Context 1. The next day, the animals are placed in Context 2 with duplicates of object A. Twenty-four hours later the animals are placed in the previous day’s location (Context 2) with objects A & B and investigation of both objects is measured. Each day consists of 5 min sessions and contexts are cleaned with 10% ETOH between each animal. Exploration is defined as sniffing or touching the object with the nose or forepaws. The task scoring involves recording of the time spent exploring an object novel to the context vs. time spent exploring an object familiar to the context, and calculation of a novelty or DI is based on these measurements and calculated as [Exploration of object B/(Exploration of object A + object B)]. Rats normally will preferentially investigate object B given that object B is a familiar object that is now presented in a novel context. NOIC is hippocampal dependent, as performance is impaired in rats with reversible muscimol-induced inactivation of the dorsal HPC CA1^15^.

### Spatial working memory

Spatial working memory is a component of short-term memory involving encoding and remembering information about one’s environment and spatial orientation. The spatial working memory task used in the present study involves an elevated white circular Barnes maze (Diameter: 122 cm, Height: 140 cm) with 18 holes evenly spaced around the outer edge of the table’s circumference. A hidden black escape box (38.73 cm × 11.43 cm × 7.62 cm) is placed under one of the holes. There are four sets of visuospatial cues (black and white stripes, a white circle, a red triangle, and an assortment of irregular shapes) placed on each of the walls surrounding the table. One day after the habituation session, spatial working memory training begins. Rats utilize visuospatial cues to learn the location of the escape box, while being exposed to mildly aversive stimuli (120 W bright overhead light, 75 db white noise), which motivates them to find the escape box to avoid these stimuli^63^. Each rat receives two trials per day for five training days. The two trials are separated by 2 min (during which the maze is cleaned and rotated). Importantly, the escape box is placed in the same location for both trials that occur on each individual training day, but is placed in a new location at the beginning of the first trial for each subsequent training day. Errors are measured as investigation of holes that do not contain the escape box, and spatial working memory is measured as the difference in the number of errors from trial 2 to trial 1 on each separate training day (which is then averaged over the five training days). Control rats (Controls, Fig. 2b below) show improved performance (fewer errors) on the second trial of each training day compared to the 1^st^, indicating that the spatial location of the escape hole can be integrated into working memory capacity in control rats.

### Deprivation intensity discrimination

Deprivation intensity discrimination involves learning to use different levels of food restriction as interoceptive discriminative stimuli for sucrose reinforcement. The behavioral paradigm follows that from previous publications^6, 31, 64^. Training: Rats are divided into Group 0 + or Group 24 + and food deprivation levels alternate each day between 0 and 24 h for the entire experiment. Group 0 + receives a reinforcement of five sucrose pellets (45 mg, Product F06233, Bio-Serv) at the end of 4 min training sessions that take place under 0 h food deprivation, and receive no pellets during training sessions that take place under 24 h deprivation. Group 24 + receives the opposite contingency between food deprivation level and pellet delivery. During sessions in which rats were trained under their non-rewarded deprivation condition, the feeders operated but no pellets were delivered. The rats were then given 2 min to consume the pellets before being removed from the conditioning chambers and returned back to their home cages. Training sessions always occurred at the same time of day (10:00 h), but not every day to avoid a single-alternating schedule of pellet delivery. The index of learning in the deprivation intensity discrimination task is food-anticipatory responding (head pokes in sucrose pellet delivery location; detected with photobeam interruptions/magazine entries) during the 1 minute prior to pellet delivery. More specifically, the mean of the percentage of the three 20 s intervals during the last minute of the session in which the magazine photobeam was interrupted was calculated as the dependent variable (as we’ve previously described^6^). Rats with HPC lesions are impaired in learning and retention of this discrimination problem^6, 65^, indicating that the HPC is critical for this type of learning process. Training consisted of six eight-trial blocks (eight trials for each deprivation state per training block), and testing (same procedures as training) consisted of one eight-trial block 7 days after surgical recovery.

### Social transmission of food preference

STFP learning involves the utilization of olfactory cues experienced during a social encounter to guide subsequent preference for a scented food. STFP task procedures were adapted from^16^. Briefly, demonstrators (non-treated rats) and observers (surgicated rats, controls and experimental) are first habituated to an unscented powdered rodent chow [LabDiet 5001 (ground pellets)] overnight. Twenty-four hours later, observers are then assigned to demonstrators (1 demonstrator assigned for 2 observers, counterbalanced by observer group) and are habituated to social interaction by being placed in a social interaction arena (23.5 cm W × 44.45 cm L × 27 cm H; clear plastic bin with Sani-chip bedding covering the bottom) to interact with each other for 30 min. The next day following a 23 h period of food restriction for all rats, demonstrators are given access to one of two flavors of powdered chow (flavored with 2.5% marjoram or 0.5% thyme; counterbalanced) for 30 min in a separate room from observers. Consistent with previous published work^66^, our pilot studies demonstrated that animals equally prefer these flavors of chow. The demonstrator is then immediately placed in the social interaction arena with one of their two assigned observer rats and allowed to socially interact for 30 min, followed immediately by a 30 min interaction with the second assigned observer rat (observer rat order counterbalanced across experimental groups). Immediately after social interactions, observers are then returned to their home cages and allowed to eat ad libitum for 1 h. Twenty-four hours later, the 23 h food-restricted observers are tested for STFP by placing two jars in the home cage: one containing paired flavor (flavored chow that was given to the demonstrator) and the other with non-paired flavor (novel flavored chow not given to the demonstrator animal). Rats are then allowed to eat for 1 h and % preference for the paired flavor is calculated by: 100*Demonstrator-paired flavored chow intake/Demonstrator + Novel flavored chow intake. Cumulative food intake for paired and non-paired flavors are calculated. Untreated control animals learn to prefer the demonstrator-paired flavor based on social interaction and olfactory food cues from the breath of the demonstrator rat; however, lesioning the dorsal HPC impairs consolidation of this preference learning.

### Zero maze

Seven days following memory test procedures, all groups of rats (SDV, sham, CCK-SAP, and SAP) were tested in the zero maze task to examine anxiety-like behavior. The zero maze is an elevated circular track, divided into four equal length sections. Two sections were open with 3 cm high curbs, whereas the two other closed sections contained 17.5 cm high walls. Animals were placed in the maze for 5 min before being returned to the home cage. After the trial, the maze was cleaned with 10% ethanol. Innate anxiety was measured as the number of open section entries and total time spent in open sections, defined as the head and front two paws in open sections. Previous studies have demonstrated that animals with high levels of innate anxiety (based on anxiolytic and anxiogenic experimental manipulations) spend less time in the open sections compared with animals with low levels of innate anxiety^67^.

### Open field

Three days following zero maze procedures, the CCK-SAP rats were then tested in the open field task, another behavioral paradigm used in rodents to evaluate innate anxiety-like behavior^67^. Open field procedures are derived from^42^. The apparatus consists of a gray Plexiglas arena (60 cm × 56 cm), and a designated center zone within the arena (19 cm × 17.5 cm), placed under diffused lighting (30 lux). Animals are placed in one of the bin’s four corners and allowed to freely explore for 10 minutes. The apparatus is cleaned with 10% ethanol between rats. Innate anxiety was measured as the number of entries into the center zone, distance moved in center zone, and total distance moved in the entire arena. Previous work has shown that animals with innate anxiety-like behavior will spend less time in the center zone and more time in close proximity to the walls compared with animals with low levels of innate anxiety^67^.

### c-Fos protein and mRNA expression

Nonsurgicated rats (*n* = 11) received i.p. injections of either saline (*n* = 5) or CCK (*n* = 6) (CCK-8, 8 μg/kg; Bachem), 90 min before perfusion and tissue was collected and processed as described above for IHC and FISH processing. IHC detection of c-Fos was performed using rabbit anti-c-Fos primary antibody (1:500, Cell Signaling, Catalog number: 2250 s) followed by a donkey anti-rabbit IgG-AlexaFluor AF488 secondary antibody (1:500, Jackson Immunoresearch, RRID: AB_2340619). In the same animals, mRNA detection followed FISH procedures (see Supplementary Methods) and used c-Fos (ACD, Catalog number: 403591), VGLUT1 (ACD, Catalog number: 317001), or GAD2 (ACD, Catalog number: 435801) probes. Representative images and quantification for c-Fos protein and mRNA expression in dCA3 and DG obtained from both i.p. saline and CCK injected animals were confined to Swanson Atlas level 28–30^68^.

### Co-injection neural tracing

Neural pathway tracing experiments utilized two tracing techniques: co-injection monosynaptic neural tracing to identify second-order relay connections (COIN)^49^ and Cre-mediated dual-synaptic anterograde tracing (below)^18^. Iontophoresis was performed using a precision current source (Digital Midgard Precision Current Source, Stoelting) as described previously^69^. Rats (*n* = 14) received unilateral iontophoretic injections of AAV1-TurboRFP (AAV1-hSyn-TurboRFP-WPRE-rBG; Penn Vector Core, Catalog number: V5574L) targeting the mNTS and, in the same animal, CTB-488 (CTB, AlexaFluor 488 conjugate; ThermoFisher, Catalog number: C22841) targeting the dCA3. Following a 12-day survival period, animals were fixation-perfused and tissue was collected and processed as described in Supplementary Methods. The native fluorescent signal was amplified using a cocktail of mouse anti-CTB (1:500, Abcam, Catalog number: ab62429) and rabbit anti-RFP (1:2000, Rockland, RRID: AB_2209751) primary antibodies followed by a cocktail of donkey anti-mouse IgG-AF488 secondary antibody (1:500, Jackson Immunoresearch, RRID: AB_2341099) and donkey anti-rabbit IgG-Cy3 (1:500, Jackson Immunoresearch, RRID: AB_2307443). Detection of AAV1 and CTB was by visualization of red (TurboRFP and Cy3) and green (AlexaFluor 488) fluorescence. In 3 of the 14 animals, injection sites were confined to the mNTS (AAV1) and dCA3 (CTB) in the same animal. Similar neuroanatomical labeling was observed in each of these three animals and representative images were obtained from one of these animals. Experimental controls (*n* = 11) were included when neither anterograde nor retrograde labeling was observed in the MS following injection sites adjacent to (respectively) the mNTS or dCA3 that did not include these regions.

### Cre-mediated multisynaptic anterograde tracing

Rats (*n* = 16) received a unilateral iontophoretic co-injection of AAV-Cre (4:5; AAV1-hSyn-Cre-WPRE-hGH; Penn Vector Core, Catalog number: CS1087) and, to determine injection site, CTB-488 (1:5; ThermoFisher, Catalog number: C22841) in the mNTS. Next, the rats received a 200 nl pressure injection of AAV-Flex-tdTomato (diluted 1:2 in 0.1 M sodium phosphate-buffered saline, pH 7.4, AAV1-CAG-Flex-tdTomato-WPRE-rBG; Penn Vector Core) in the MS. Following a 3-week survival period, animals were perfused and tissue was collected and processed as described in Supplementary Methods. AAV1-Flex-TdTomato and CTB-488 was detected based on a combination of native fluorescence of RFP and green fluorescent protein, respectively, and amplification of signal using the same cocktail of primary and secondary antibodies as described in the co-injection neural tracing section. Representative images were obtained from one of the three animals with both CTB-488 and AAV1-Flex-tdTomato injection sites that were predominantly confined to the mNTS (CTB) and MS (AAV1), respectively (similar labeling was observed in the other two animals that were double hits). The specificity of this connection was further supported by the absence of axon labeling in dCA3 and DG following MS injections of AAV-FLEX-TdTomato alone (*n* = 3; w/AAV2/1-hSyn-Cre injections adjacent to mNTS), mNTS injections of AAV2/1-hSyn-Cre alone (*n* = 4; w/AAV-FLEX-TdTomato injections adjacent to the MS), or in rats in which either injection site was off target (misses, *n* = 6).

### Statistical analyses

Terminal body weights and zero maze performance data were analyzed using one-way ANOVA, with surgery group as a between-subjects factor for both SDV and CCK-SAP groups. NOIC was analyzed using repeated-measures ANOVA with surgical group (between-subjects) and testing day (within-subjects) as factors for both SDV and CCK-SAP experiments, with Newman–Keuls post hoc comparisons to evaluate group differences on each separate day. Spatial working memory data (Barnes maze) were analyzed using repeated-measures ANOVA across training days with surgical group as a between-subjects variable and training day as a within-subjects variable. The deprivation intensity discrimination test data were analyzed using repeated-measures ANOVA with surgical and deprivation (0 + , 24 + ) assignment group as between-subjects factors, with deprivation state as a within-subjects factor, and Newman–Keuls post hoc tests for individual group × deprivation state comparisons. The STFP percent paired flavor preference data was analyzed using one-way ANOVA with surgical group as a within-subjects factor. Postmortem immunoblot for both SDV and CCK-SAP groups were analyzed using ANOVA with surgical group as a between-subjects factor. Linear regressions were used to calculate p-values, *R*^2^ goodness-of-fit, 95% confidence bands of the best-fit line, and linear equations between NOIC and Barnes data with protein level data by surgical group. All groups (SDV, CCK-SAP, and their controls) were analyzed for linear regression of HPC protein expression vs. Barnes performance, whereas only CCK-SAP and their controls were analyzed for HPC protein expression vs. NOIC performance (as immunoblot HPC tissue was not extracted from SDV and sham/controls that underwent NOIC testing). Normality was confirmed for all analyses using Shapiro–Wilk’s test. c-Fos protein and mRNA expression levels in dCA3 and DG were analyzed using one-way ANOVA with drug treatment group as a between-subject factor. All statistical analyses were performed using the statistical software Statistica (Version 7; Statsoft) and linear regressions analyses were performed using Prism 7 (GraphPad) statistical software. Sample size was chosen based on a priori power analyses (conducted in Statistica V7) to ensure sufficient power to detect a pre-specified effect size. Pre-established exclusion criteria used was the Grubbs test for outliers (conducted in Prism 7). ANOVAs were followed by Newman–Keuls post hoc comparisons when significant main effects or interactions were obtained. Results are presented as mean ± SE. Statistical significance was set at *p* *<* 0.05.

### Data availability

The data that support the findings of this study are available in the Open Science Framework Repository [10.17605/OSF.IO/UYMBQ]^70^.

## Electronic supplementary material

Supplementary Information
