# Supplementary material for: Gut vagal sensory signaling regulates hippocampus function through multi-order pathways
Source: Nat Commun. 2018 Jun 5;9:2181. doi: 10.1038/s41467-018-04639-1 (PMC5988686; doi:10.1038/s41467-018-04639-1)
Supplement: Supplementary file 1 — Supplementary Information [file 41467_2018_4639_MOESM1_ESM.pdf]

Gut vagal sensory signaling regulates hippocampus function through multi-order pathways

Suarez et al.

Supplementary Methods

Immunoblotting

Within 5-6d following the last behavioral procedure, rats (sham [n=7], SDV [n=8], sap [n=8], CCK-SAP [n=9] groups) were anesthetized with a ketamine (90 mg/kg), xylazine (2.7 mg/kg), and acepromazine (0.64 mg/kg) cocktail and brains were removed from the skull after decapitation and placed on a brain matrix (Kopf Instruments) for coronal sectioning (2mm sections). Tissue were rapidly extracted bilaterally from one 2mm thick section using a 2mm inner diameter tissue punch. The brain tissue was immediately flash frozen in cooled isopentane and stored in -80°C until further processing. Proteins in brain lysates were separated using sodium dodecyl sulfate polyacrylamide gel electrophoresis, transferred onto poly-vinylidene difluouride membranes, and subjected to immunodetection analysis using enhanced chemiluminescence (Chemidoc XRS, BioRad). A rabbit anti-brain-derived neurotrophic factor antibody (1:500, Santa Cruz Biotechnology, Catalog # sc-20981) was used to evaluate the concentration of BDNF relative to a loading control signal detected by a rabbit anti-beta actin antibody (1:5000, Santa Cruz Biotechnology, Catalog # NB600-503). A rabbit anti-doublecortin antibody (1:500, Abcam, Catalog # ab18723) was used to evaluate the concentration of doublecortin (DCX) relative to a loading control signal detected by an anti-b tubulin antibody (1:5000, Cell Signaling, Catalog #2128S). Blots were quantified with densitometry analysis using Image J as described [1, 2, 3].

Immunohistochemistry

Animals were anesthetized, then transcardially perfused with 0.9% saline, followed by 4% paraformaldehyde in 0.1 M borate buffer (pH 9.5). Brains were removed and immersed in 12% sucrose in PFA fixative for 24 h at 4°C. The brains were then flash-frozen in dry-ice cooled isopentane before sectioning on a sliding microtome at 30 µm. Sections are stored in antifreeze solution at -20°C until immunohistochemistry (IHC) processing. During IHC procedures, antibody incubations were done at 4°C (washes and other steps at room temperature). Tissues were permeabilized in 0.3% Triton-X (Triton-X 100, Sigma, Cat #: X100-500ML) for 30 min and were blocked in 2% normal donkey serum (Donkey serum, Jackson Immunoresearch, Cat #: 017-000-121) for 10 min. Sections were incubated in primary antibodies overnight (~18 hrs). Primary antibodies were diluted in the following solution: KPBS containing 2% normal donkey serum. After incubation in primary antibodies, the sections were washed in KPBS (8 changes, 10 min each). Secondary antibody incubations were performed overnight. Secondary antibody-fluorophore conjugates were diluted in KPBS + 2% donkey serum. After secondary antibody incubation, the sections were washed in KPBS (6 changes, 10 min each). The sections were then mounted onto glass slides, air dried, and coverslipped with 50% glycerol in KPBS mountant. Photomicrographs were acquired using either a Nikon 80i (Nikon DS- QI1,1280X1024 resolution, 1.45 megapixel) under epifluorescent illumination using Nikon Elements BR software.

Fluorescent in situ hybridization

Animals were perfused and tissue was harvested and processed as described above in IHC methods. FISH processing procedures followed those previously described [4]. Tissue

sections were mounted on subbed glass slides (Fisher brand Superfrost Plus) and placed in a vacuum desiccant chamber overnight (~16 hrs). Slides were postfixed in 4% PFA for an hour and 45 minutes, then washed 5 x 5 minutes in KPBS. Slides were incubated for 30 min at 37°C in a solution of 100mM Tris (pH 8), 50mM EDTA (pH 8), and 0.1% Proteinase K (10mg/mL, Sigma P2308) in water, and then rinsed for 3 min in the same Tris and EDTA solution without Proteinase K. Slides were rinsed for 3 min in a solution of 100mM triethanolamine (pH 8) in water, and then incubated for 10 min at room temperature with 0.25% acetic anhydride in 100mM triethanolamine. Slides were rinsed twice at 2 min each in a solution of 10% 20X saline-sodium citrate buffer. Slides were then dehydrated in increasing concentrations of ethanol (50%, 70%, 95%, 100%, 100%) and air dried before proceeding to hybridization. The probes were applied and incubated for 3 hours. Reagents from the RNAscope® Fluorescent Multiplex Detection Reagent Kit (ACD, Cat #: 320851) were used to amplify the probe, with AMP 1 applied for 45 minutes, AMP 2 for 30 minutes, AMP 3 for 45 minutes, and AMP 4 for 30 minutes. Between and after each incubation the slides were washed in RNAscope® Wash Buffer 1X (ACD, Cat #: 320058) twice for 2 minutes each. Slides were coverslipped using ProLong® Gold Antifade Reagent (Cell Signaling, Cat #: 9071S). Photomicrographs were acquired using either a Nikon 80i (Nikon DS- QI1,1280X1024 resolution, 1.45 megapixel) under epifluorescent illumination using Nikon Elements BR software.

Supplementary Fig. 1

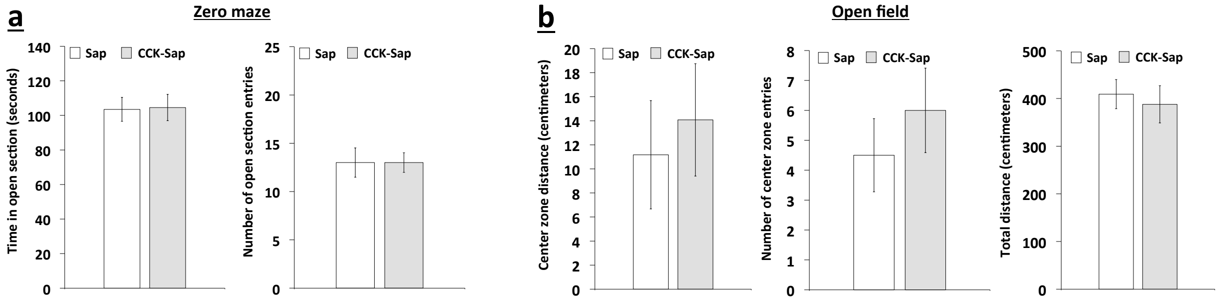

Supplementary Fig. 1: CCK-SAP-mediated vagal GI afferent ablation does not impact anxiety-like behaviors. (**a**) Time spent in open arm section (seconds) (ANOVA, F[1,15]=0.0103, p=0.92) and number of open section entries (ANOVA, F[1,15]=0, p=1.0) during zero maze anxiety test for the CCK-sap (n=9) vs saporin (n=8) groups. (**b**) Center zone distance (centimeters) (ANOVA, F[1,15]=0.198, p=0.663), number of center zone entries (ANOVA, F[1,15]=0.6269, p=0.441), and total distance (centimeters) (ANOVA, F[1,15]=0.1784, p=0.679) during open field test for the CCK-sap (n=9) vs saporin (n=8) groups. (*P<0.05 vs saporin. Data are mean ± s.e.m. CCK-SAP, cholecystokinin-saporin).

Supplementary Fig. 2

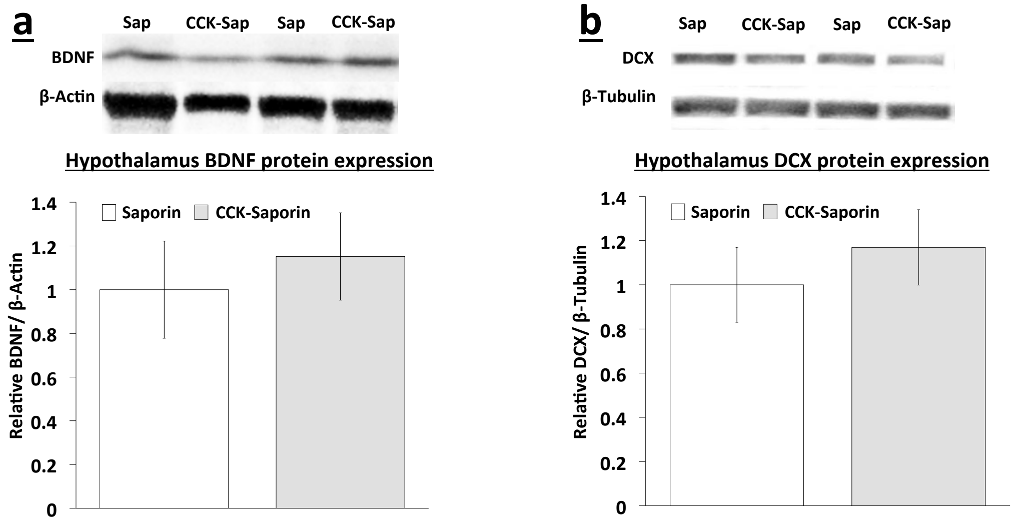

Supplementary Fig. 2: CCK-SAP does not affect neurotrophic (BDNF) and neurogenic (DCX) markers in the hypothalamus. (**a**, **b**) CCK-SAP-mediated GI vagal afferent ablation does not impact hypothalamus BDNF and DCX expression relative to SAP controls (ANOVA, $F[1,13]=0.26$, $p=0.619$ (SAP, n=7, CCK-SAP, n=8; BDNF); ANOVA, $F[1,14]=0.4995$, $p=0.493$ (SAP, n=8, CCK-SAP, n=8; DCX)). (Data are mean ± s.e.m. BDNF, brain-derived neurotrophic factor; DCX, doublecortin; CCK-SAP, cholecystokinin-saporin).

Supplementary Fig. 3

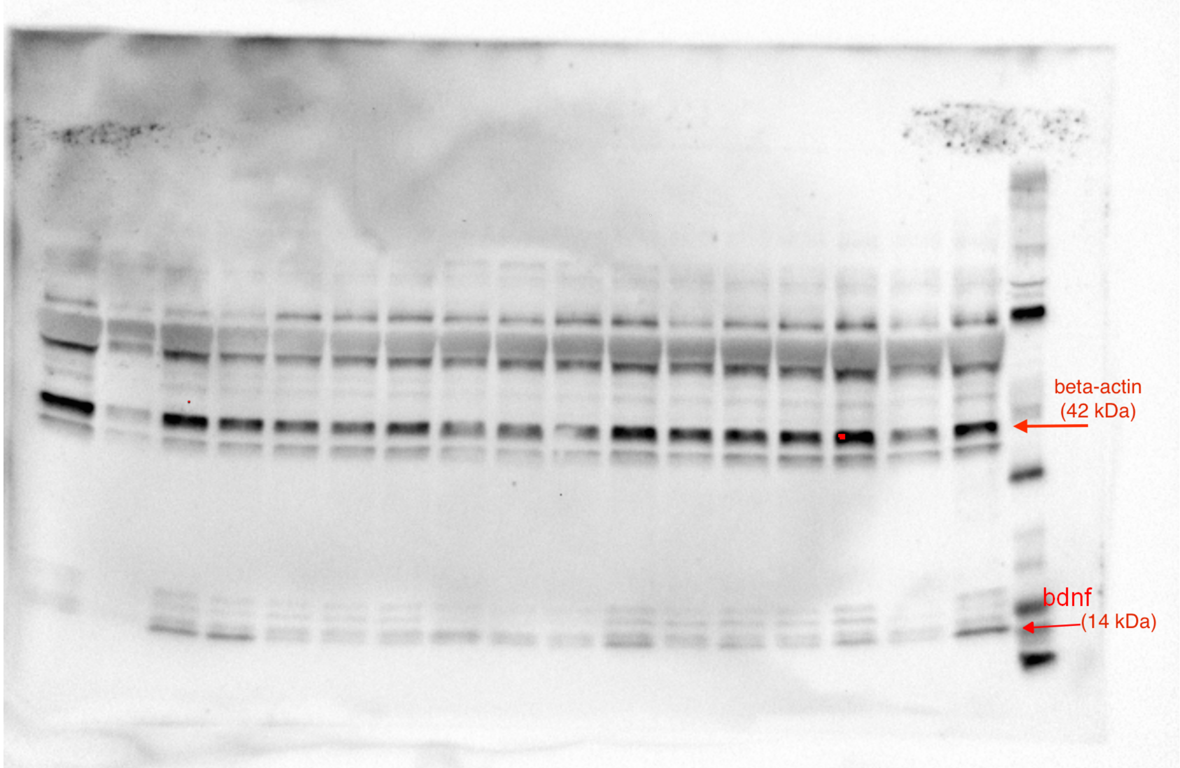

Supplementary Fig. 3: BDNF markers in the hypothalamus. Uncropped scan of hypothalamus brain-derived neurotrophic factor (BDNF; 14 kDa) and positive loading control (beta-actin; 42 kDa) in CCK-SAP vs SAP animals.

Supplementary Table 1

Group	Training deprivation level		Surgery	Testing deprivation level	
	0-hr	24-hr		0-hr	24-hr
0+	Sucrose +	Sucrose -	SDV 0+	Sucrose +	Sucrose -
			Sham 0+	Sucrose +	Sucrose -
24+	Sucrose -	Sucrose +	SDV 24+	Sucrose -	Sucrose +
			Sham 24+	Sucrose -	Sucrose +

Supplementary Table 1: Timeline of pre-surgery training and post-surgery testing for deprivation intensity discrimination paradigm. Adapted from [5]. 0+: rewarded under 0-hr but not 24-hr food deprivation; 24+: rewarded under 24-hr but not 0-hr food deprivation; Sucrose +: sucrose reward at the end of session; Sucrose -: no sucrose reward at end of session; SDV: total subdiaphragmatic vagotomy.

Supplementary References

1. Kanoski SE, Fortin SM, Ricks KM, Grill HJ. Ghrelin signaling in the ventral hippocampus stimulates learned and motivational aspects of feeding via PI3K-Akt signaling. *Biol Psychiatry* **73**, 915-923 (2013).

2. Kanoski SE, Ong ZY, Fortin SM, Schlessinger ES, Grill HJ. Liraglutide, leptin and their combined effects on feeding: additive intake reduction through common intracellular signalling mechanisms. *Diabetes Obes Metab* **17**, 285-293 (2015).

3. Hsu TM*, et al.* Effects of sucrose and high fructose corn syrup consumption on spatial memory function and hippocampal neuroinflammation in adolescent rats. *Hippocampus* **25**, 227-239 (2015).

4. Hsu TM*, et al.* A hippocampus to prefrontal cortex neural pathway inhibits food motivation through glucagon-like peptide-1 signaling. *Mol Psychiatry*, (2017).

5. Davidson TL, Kanoski SE, Chan K, Clegg DJ, Benoit SC, Jarrard LE. Hippocampal lesions impair retention of discriminative responding based on energy state cues. *Behav Neurosci* **124**, 97-105 (2010).
